# Synchronized Data Collection for Human Group Recognition †

**DOI:** 10.3390/s21217094

**Published:** 2021-10-26

**Authors:** Weiping Zhu, Lin Xu, Yijie Tang, Rong Xie

**Affiliations:** School of Computer Science, Wuhan University, Wuhan 430072, China; cathyxl@outlook.com (L.X.); tangyijie@whu.edu.cn (Y.T.); xierong@whu.edu.cn (R.X.)

**Keywords:** group recognition, synchronization, trajectory interpolation, message passing

## Abstract

It is commonplace for people to perform various kinds of activities in groups. The recognition of human groups is of importance in many applications including crowd evacuation, teamwork coordination, and advertising. Existing group recognition approaches require snapshots of human trajectories, which is often impossible in the reality due to different data collection start time and frequency, and the inherent time deviations of devices. This study proposes an approach to synchronize the data of people for group recognition. All people’s trajectory data are aligned by using data interpolating. The optimal interpolating points are computed based on our proposed error function. Moreover, the time deviations among devices are estimated and eliminated by message passing. A real-life data set is used to validate the effectiveness of the proposed approach. The results show that 97.7% accuracy of group recognition can be achieved. The approach proposed to deal with time deviations was also proven to lead to better performance compared to that of the existing approaches.

## 1. Introduction

People often perform various kinds of activities in groups. For example, friends, colleagues, or a family often go shopping together [1,2], firefighters often form several groups to search survivors in a burning building [3,4], and people in an earthquake often escape with familiar persons [5]. It was reported that up to 70% of people’s time in public places is spent with others [6]. High cohesion of a group enables more effective information dissemination and human management. For example, if escape instructions are sent to human groups rather than individuals in an emergency situation, the redundant message transmission can be avoided, and thus, leads to a faster evacuation.

Existing works on the recognition of human groups mainly perform spatial-temporal clustering or collective matrix factorization based on the snapshots of people’s trajectory [2,7,8,9,10]. The snapshots of people’s trajectory are critical to the human group recognition. One of the challenges is that data are required to be collected at the same time from different persons. We call this problem the synchronization of group data collection. There are many factors affecting it because the processing is inherently distributed in different sensing devices carried by people. Firstly, the sensing devices may start their data collections at different times. Secondly, the data collection frequencies are different because there are different parameter settings in sensing devices. Thirdly, the clocks at different sensing devices are usually not well synchronized. The synchronization usually incurs high overhead, and sometimes is even impossible because of hardware constraints or privacy concerns. What is worse is that the degree of the deviation of a clock to other clocks is not known. Due to the aforementioned reasons, it is difficult to collect data from people at the same time. Further data processing is demanded to improve this situation.

This study focuses on the synchronized data collection for human group recognition. Firstly, we synchronize the data of different people by interpolating data at a certain period from a starting time. Detailed algorithm to compute the optimal starting time is also illustrated. After that, we estimate the deviation of clocks between sensing devices based on message passing. An approach is proposed to minimize such deviation using limited number of message passings. Finally, we use a real-life data set to evaluate the proposed approaches. In summary, this study makes the following contributions:

- We identified the synchronized data collection problem in the human group recognition.
- We proposed a trajectory interpolation algorithm to solve different start time and frequency problem in the human group recognition. A reasonable error function is designed to optimize the interpolation.
- We utilize message passing to estimate and minimize the deviation of clocks between devices.
- Extensive evaluations are carried out and the results show that the proposed algorithms outperform the existing approaches.

The rest of the paper is organized as follows: Section 2 reviews the related works. Section 3 introduces the system model of this study. Section 4 illustrates the aligned trajectory interpolating algorithm to deal with different start times and frequencies. Section 5 illustrates the approach to estimate and minimize the deviation of clocks between sensing devices. Section 6 reports the evaluation results, and Section 7 concludes this paper.

This study is based on our previous study [11]. In this version, we consider the time deviations among devices as an important factor to affect the accuracy of human group recognition, and propose an approach to estimate and eliminate such time deviations.

## 2. Related Work

In recent years, many researchers investigated the group recognizing based on sensors. Wirz [12] et al. proposed a pedestrian flock detection algorithm by using the spatial-temporal clustering [13] of a series of snapshots of GPS data of humans. Density-based clustering algorithms such as DBSCAN [14] and DJ-Cluster [15] can be used for their clusterings. The works [16,17] follow the similar idea based on GPS positioning data. The similarity of other sensing signals including acceleration, orientation, WiFi signals, and Bluetooth signals [1,18,19] can also be used to deduce the groups. Feese [3,4] et al. detected groups of firefighters by using the built-in ANT radio and atmospheric pressure sensor of mobile phones. The ANT radio based communications are used to determine the distance between two firefighters, and the atmospheric pressure sensor is used to determine their located floors in a building. These data are further combined to detect the groups of moving firefighters. Shen et al. utilize the similarities of RSS trends of WiFi data from people to infer their group affiliations [20]. They further combine the smartphone usage behaviors (measured by the number of bursts of WiFi probes) into the computation of similarities [2]. More features including the spatial features, signal strength features, motion features, turn features, and level change features of humans are combined to deduce the group affiliation [1,9,21,22,23]. One of our previous works utilizes the interaction among humans to improve the recognition accuracy [24]. Instead of using raw sensing data, the works [25,26] proposed a group affiliation detection algorithm based on the data distribution, i.e., a mixture of Gaussian for acceleration and a mixture of von Mises distributions for orientation. This method greatly reduces the amount of data to be transmitted, and hence, gains benefits in the real-time processing and energy consumption. All these works assumed that the sensing data are synchronized and collected in snapshots. However, this assumption is not always satisfied. This study solves this problem.

There are also existing works for synchronizing the clocks among devices, including server-slave communization-based approaches [27,28], broadcasting (single-hop or multihops) based approaches [29], and hierarchical communization-based approaches [30,31]. The natural network effect and more complex models are also used to improve the synchronizing accuracy [32,33,34]. However, in many scenarios, implicit synchronization is impossible due to hardware constraints of devices or privacy concerns from people. In this study, we use the communications in the application layer to avoid this problem.

## 3. System Model

We assume that in a region such as a shopping mall there are *n* persons forming multiple groups to perform activities. Each person’s data are collected by their mobile devices or the sensors installed in the region. The data are assumed collected with a duration of *T*. Data collection starts when people come into the region. People may come into the region at different times, which causes different start times of data collection. Person *i*’s data are collected periodically with a period of $F_{i}$. Therefore, we obtain a sequence of location data $P_{0}$, $P_{1}$, …, $P_{n-1}$ where $P_{i}$ is the location data for person *i* (0≤i<n). The clocks of the data collection devices are not calibrated so inherent time deviations exist. The different data collection start time, frequency, and time deviation causes unaligned collected data.

**Figure 1:**
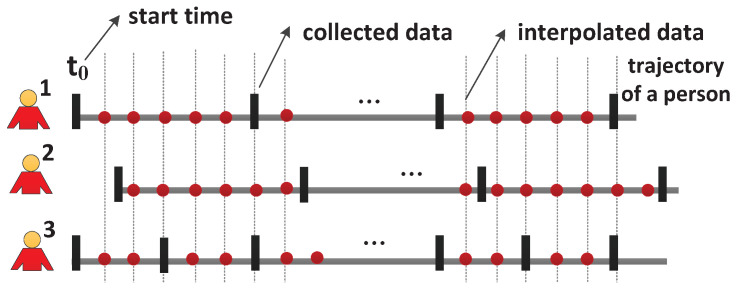
Data collection process of human groups. Rectangles denote timestamps when data are collected, and dots denote timestamps when data are interpolated. Collected data are not aligned, but interpolated data are well aligned.

We use Figure 1 to demonstrate the different start times and frequencies in the data collection process. The rectangles represent the timestamps of data collection. According to the figure, person 1 and person 2 start their data collections at different times, and thus the collected data are not aligned. Person 1 and person 3 have different data collection frequencies, and thus, also have unaligned collected data. The unaligned data make group recognition difficult because group recognition is based on the snapshots of the states of persons.

Our purpose is dual fold. Firstly, we aim to synchronize the data collection assuming that all clocks have no time deviations. Data interpolation technique can be used to achieve this purpose. As displayed in Figure 1, the dots represent the interpolated data, which are aligned for all persons. After the interpolation, all the data used for group recognition start at the time $t_{0}$ and with the period of *f* where $f\leq \min F_{i},\left(i=0,1\ldots n-1\right)$. The determining of *f* depends on the requirement of group recognition application (e.g., online update frequency). We determine the interpolation approach and the optimal $t_{0}$. Secondly, we aim to estimate the time deviation among devices and further align the collected data. The messages exchanged among devices can be used to compute the time deviation. Since different messages have different impact on the time deviation estimation, we determine the optimal messages to be sent.

## 4. Aligned Trajectory Interpolation

We first introduce the aligned trajectory interpolation approach for synchronizing the collected data. This approach follows Catmull–Rom Spline [35] algorithm to interpolate a datum in the middle of four consecutive data (called control points). It holds the property that the control points are in the B$\overset{´}{e}$zier curve and no cusp and intersection on the curve determined by the control points and interpolated data [35], which is consistent with the human’s walking trajectory.

The interpolation process include two steps as follows:

(a) Add one datum (i.e., $2P_{0}-P_{1}$) before the first collected datum (i.e., $P_{0}$), and one datum (i.e., $2P_{n}-P_{n-1}$) after the last collected datum (i.e., $P_{n}$). This is to guarantee that there are at least four collected data.

(b) Go through the whole data set, obtain four data each time as control points, and interpolate one datum between the middle two control points. The interpolation is performed at $t_{i}=t_{0}+i*f$ ($0\leq t_{0}<f$, i=1,2…).

Following a proper starting time $t_{0}$ and period *f*, the interpolated data can be aligned. Assuming that [1, 2, 3] and [1.3, 2.3, 3.3] are two collected data, if $t_{0}$ is 0 and *f* is 0.3, the resulting data [1, 1.3, 1.6, 1.9, 2, 2.3, 2.6, 2.9, 3], [1.3, 1.6, 1.9, 2.2, 2.3, 2.6, 2.9, 3.2, 3.3] are aligned.

The value of starting time $t_{0}$ affects the accuracy of the interpolation. We further define an error function to measure the difference between the interpolated data and the real data. We aim to obtain the data close to the real ones by minimizing the error function. To design the error function is quite challenging. Empirically, interpolation error of one datum becomes larger when it is farther away from the control points. Based on this idea, we propose an error function as follows:

(1) $$g\left(x\right)=-10{\left(1-x\right)}^{10}+10{\left(1-x\right)}^{9}$$

where x∈[0,1] represents the normalized distance of the interpolated datum from the second control point to the third control point.

With the error function being determined, given a $t_{0}$, we can compute the error of each interpolated datum and accumulate it to the total errors. By iterating $t_{0}\in \left[0,f\right)$, we can obtain the optimal value of $t_{0}$ such that the total errors are minimized. The detailed algorithm is illustrated in Algorithm 1. We assume that all the user data are stored in *userData*, where *userData*${}_{i}$ represents the data of user *i*. *interpolatedData*${}_{i}$ stores all the interpolated data of user *i*. $t_{0}$ is checked with a time unit *unitTime*. We first obtain all the interpolated data as in lines 5–7. Then, for each interpolated datum, we find its two nearest control points ($d_{0}$ and $d_{1}$), and compute the normalized distance of it from $d_{0}$ to $d_{1}$ (lines 8–9). Based on the error function of Equation (1), we can compute the error of this interpolated datum, and accumulate it into *errors* (line 10). We iterate all possible values of $t_{0}$ in [0, *f*) and the optimal value is the one that achieves the minimal errors (lines 3 and 13–14).
**Algorithm 1:** Start time determination
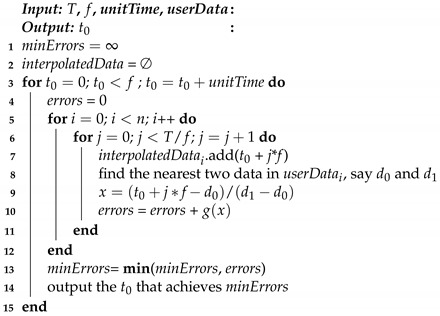


**Figure 2:**
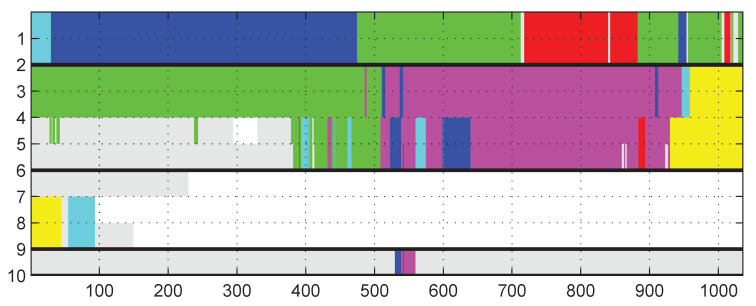
Graphical display of human groups. Persons with ID 1–10 are grouped from timestamp 1–1000. Persons in one group are marked with same color. Blank space means no data, and gray space means that person currently does not belong to any group.

After the data interpolation, we follow Wirz’s spatial-temporal flock detection algorithm [12] to recognize the human groups. In each snapshot, the algorithm divides all the people into groups by DJ-Cluster [15], according to people’s locations. In this approach, we use DBSCAN [14] rather than DJ-Cluster, because DBSCAN can detect a complete cluster more fast for real-time processing. After that, the deduced groups are compared with the ones identified in previous snapshots. If two groups in continuous snapshots are sufficiently similar, they are regarded as the same group. Real-time graphically monitoring of the groups is also feasible in this situation. An example of it is shown in Figure 2.

## 5. Time Deviation Estimation and Elimination

In the previous section, we assume that the clocks among all the devices are synchronized. Actually, this assumption is not always satisfied. The time deviation among the devices commonly exists and affects the accuracy of group recognition. In this section, we try to minimize the time deviation among the devices.

### 5.1. Time Deviation Estimation Based on Message Passing

**Figure 3:**
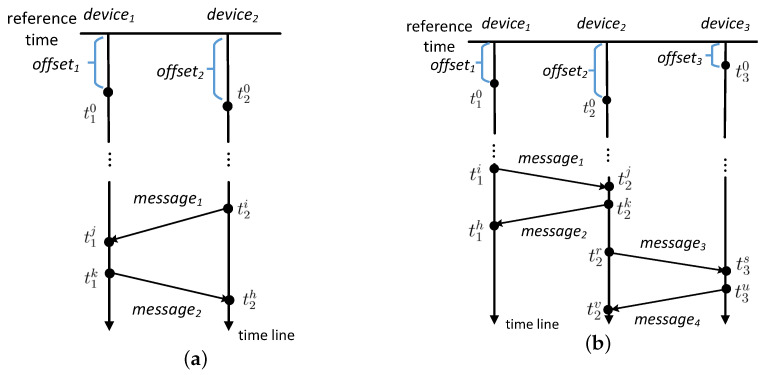
(**a**) *device${}_{1}$* and *device${}_{2}$* has a bidirectional message. (**b**) *device${}_{1}$* and *device${}_{3}$* has an indirect bidirectional message through *device${}_{2}$*.

We first estimate the time deviation of devices before eliminating it. Message passing can be used for this purpose. Message passing is a technique to determine the temporal relations among devices without synchronized clocks. The main idea is based on the fact that the sending timestamp of a message must be prior to its receiving timestamp [36]. We bound the time deviation of devices based on message passing. Figure 3a illustrates how message passing works where *device*${}_{1}$ and *device*${}_{2}$ have *offset*${}_{1}$ and *offset*${}_{2}$ deviated to a reference timestamp, respectively. These devices transmit messages to each other where each message passing is denoted by an arrow (e.g., *message*${}_{1}$ and *message*${}_{2}$). We have the following inequalities:

$t_{2}^{i}$ + *offset*${}_{2}$ < $t_{1}^{j}$ + *offset*${}_{1}$

$t_{1}^{k}$ + *offset*${}_{1}$ < $t_{2}^{h}$ + *offset*${}_{2}$

By solving the inequalities, we obtain the time deviation of the two devices *offset*${}_{1}$−*offset*${}_{2}$∈ [$t_{2}^{i}-t_{1}^{j}$, $t_{2}^{h}-t_{1}^{k}$]. A message from *device*${}_{1}$ to *device*${}_{2}$ determines the upper bound of *offset*${}_{1}$−*offset*${}_{2}$ while a message from *device*${}_{2}$ to *device*${}_{1}$ determines the lower bound of *offset*${}_{1}$−*offset*${}_{2}$. A bidirectional message determines the time interval for *offset*${}_{1}$−*offset*${}_{2}$. The length of the time interval is the sum of the two messages’ transmission durations.

Besides the aforementioned direct bidirectional messages, Figure 3b shows an indirect bidirectional message. *device*${}_{1}$ and *device*${}_{3}$ have no direct message passing, but through *device*${}_{2}$ we can determine their temporal relations. We calculate the time deviation between them using the following inequalities:

$t_{1}^{i}$ + *offset*${}_{1}$ < $t_{2}^{j}$ + *offset*${}_{2}$

$t_{2}^{k}$ + *offset*${}_{2}$ < $t_{1}^{h}$ + *offset*${}_{1}$

$t_{2}^{r}$ + *offset*${}_{2}$ < $t_{3}^{s}$ + *offset*${}_{3}$

$t_{3}^{u}$ + *offset*${}_{3}$ < $t_{2}^{v}$ + *offset*${}_{2}$

By solving the inequalities, we obtain *offset*${}_{1}$−*offset*${}_{2}$∈ [$t_{2}^{k}-t_{1}^{h}$, *t${}_{2}^{j}-$t${}_{1}^{i}$*], *offset*${}_{2}$−*offset*${}_{3}$∈ [*t${}_{3}^{u}-$t${}_{2}^{v}$*, $t_{3}^{s}-t_{2}^{r}$]. Combining the two time intervals, we have *offset*${}_{1}$−*offset*${}_{3}$∈ [$t_{2}^{k}-t_{1}^{h}+t_{3}^{u}-t_{2}^{v}$, $t_{2}^{j}-t_{1}^{i}+t_{3}^{s}-t_{2}^{r}$]. Consequently, even though *device*${}_{1}$ and *device*${}_{3}$ do not have any message exchange directly, their time deviation can be estimated.

Given a reference device, the time deviation of other devices to it can be determined by their direct or indirect message passings. We build a direct graph *G* to compute the direct or indirect message passings, where a vertex represents a device, and an edge represents a message passing between two devices. Whether a message passing between two devices exists depends on the bidirectional reachability of the two vertices in the graph. Without loss of the generality, we use the vertex that has the largest degree as the reference device.

**Figure 4:**
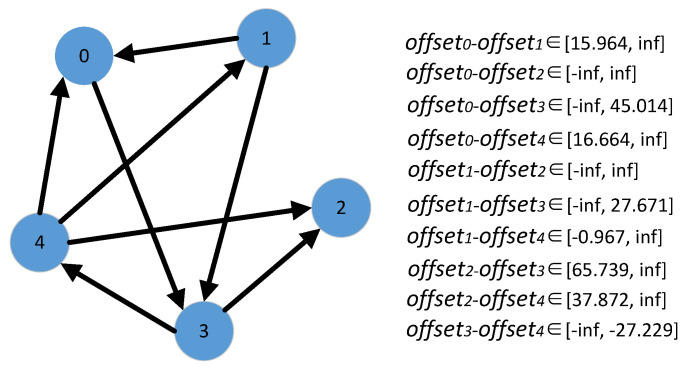
Time deviation estimation based on message passing.

Figure 4 illustrates an example of it. Vertex 3 is selected as the reference device. When determining the time deviation of vertex 4 to vertex 3, from vertex 3 to vertex 4, there is only one path 3→4, and hence *offset*${}_{3}$−*offset*${}_{4}$ is bounded by the time interval [−*infinite*, −27.229]. However, from vertex 4 to vertex 3, there are three paths, 4→0→3, 4→1→0→3, and 4→1→3, and hence *offset*${}_{3}$−*offset*${}_{4}$ is determined by the time intervals [−28.37, *infinite*], [−30.017, *infinite*], and [−28.637, *infinite*], respectively. By minimizing the length of time interval, *offset*${}_{3}$−*offset*${}_{4}$ is bounded by [−28.37, −27.229] that is derived from the paths 3→4 and 4→0→3.

Considering that initially not all the devices are bidirectionally reachable from the reference device, more message passings are required to build the temporal relations among them. Given a limited number of messages, we require to improve the estimation of time deviations of all devices to the reference device. We define two criteria to evaluate the accuracy of such estimation. One is the number of *infinites* in the time intervals bounded the time deviation, and the other is the average length of the time intervals bounded the time deviation. The number of *infinites* has the priority on the average length of the time intervals.

Table 1 illustrates the change in number of *infinites* and average length of the time intervals bounded the time deviation. This result is derived when changing the number of messages from 10 to 45 and fixing the number of devices to 10. We perform five experiments and calculate the average of them. The reduced number of *infinites* and the average length of the time intervals denote a more accurate estimation of the time deviations. For example, there exist less than two *infinites* in the time interval (average case) when the number of messages is between 10 and 20. When the number of messages is more than 25, *infinite* is gradually eliminated. The average length of the time intervals also decreases with more messages.

### 5.2. Improvement on the Estimation of Time Deviation

We use more message passings to improve the estimation accuracy of time deviations. We first compute the number of additional messages required to obtain the estimation of all the devices (i.e., eliminate all *infinites* in the estimation). Then, we discuss the sequence to add these messages to improve the accuracy speed.

The first problem can be solved by guaranteeing the strong connection of the graph *G* that represents the devices and message passings. We first calculate the least amount of edges required for the graph to become a strongly connected directed graph. The main idea for achieving this is that if one vertex is biconnected to any other vertex, the graph must be strongly connected. The detailed approach is as follows. We first follow the work [37] to detect the strongly connected components in *G* and replace each component with a new vertex (called shrinking vertex), thus obtain a new graph *G’*. After that, the in-degree and out-degree of each vertex in *G’* are computed. The least number of edges required to be added to make *G’* be a strongly connected graph is max(|VI|,|VO|) where *VI* represents the vertices whose in-degrees are 0 and *VO* represents the vertices whose out-degrees are 0. Each added edge can reduce a vertex whose in-degree is 0 and a vertex whose out-degree is 0.

For the sequence of additional message passings, we propose an algorithm to sequentially add one more message passing to improve the accuracy of time deviation estimation. The algorithm determines the start vertex and end vertex of a message passing which is helpful to reduce the number of *infinites* in the time intervals bounded the time deviations. The vertex with out-degree of 0 and the largest in-degree is selected as the start vertex, and the vertex with in-degree of 0 and the largest out-degree is selected as the end vertex. A message passing between such vertices is likely to establish more new paths in the graph, and therefore, eliminate as many *infinites* as possible and refine the time intervals bounded the time deviations.

The detailed approach is shown in Algorithm 2. The algorithm determines a message passing sent from vertex *startVertex* to vertex *endVertex*. The algorithm first computes $V_{in-degree=0}$ and $V_{out-degree=0}$ that represent the vertices whose in-degrees are 0 and the vertices whose out-degrees are 0, respectively (line 1). Then, we determine *startVertex* as a vertex in $V_{out-degree=0}$ that has the largest in-degree, and *endVertex* as a vertex in $V_{in-degree=0}$ that has the largest out-degree (lines 2 and 6). If the vertex is a shrinking vertex, the original vertex is determined as the *startVertex* or *endVertex*, following similar principles (lines 3–5 and 7–9). If $V_{out-degree=0}$ is an empty set, we choose the vertex that has the largest in-degree in G∖endVertex as *startVertex* (lines 10–12). Similarly, *endVertex* can be determined (lines 13–15).

**Figure 5:**
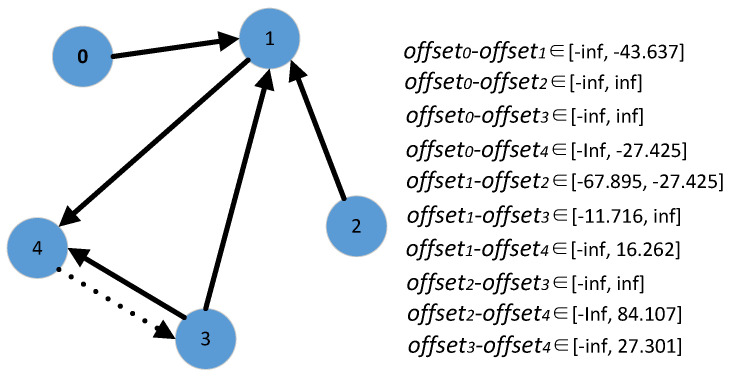
An example of additional message passing determination.

An example is shown in Figure 5 in which there are five messages initially. According to our approach, the next message to be passed should be from vertex 4 to vertex 3. By adding this message, *offset*${}_{0}$−*offset*${}_{3}$ will be bounded by [−*infinite*, −54.233], *offset*${}_{1}$−*offset*${}_{3}$ will be bounded by [−11.716, −10.596], *offset*${}_{1}$−*offset*${}_{4}$ will be bounded by [15.092, 16.212], *offset*${}_{2}$−*offset*${}_{3}$ will be bounded by [−*infinite*, 57.299] and *offset*${}_{3}$−*offset*${}_{4}$ will be bounded by [26.808, 27.301], five *infinites* can be eliminated. This message is the best one for eliminating the number of *infinites*. For example, if a message is from vertex 1 to vertex 3, *offset*${}_{0}$−*offset*${}_{3}$ will be bounded by [−*infinite*, −54.501], *offset*${}_{1}$−*offset*${}_{3}$ will be bounded by [−11.716, −10.864], and *offset*${}_{2}$−*offset*${}_{3}$ will be bounded by [−*infinite*, 57.031]. There are three *infinites* that can be eliminated.

After obtaining the time interval bounded the time deviation of a device to the reference device, we use the middle as its time deviation. This time deviation can be eliminated by adjusting the timestamps of the data collected.

The time deviation estimation is affected by the transmission time of messages and the messages in the application layer. Less transmission time leads to more accurate upper bound and lower bound of time deviation between two devices. The messages among devices in the application layer also can help to estimate time deviation, and then less independent messages are required for building the relations among devices. If an application has frequent interactions among devices, our approach can be benefit from them.
**Algorithm 2:** Additional Message Passing Determination
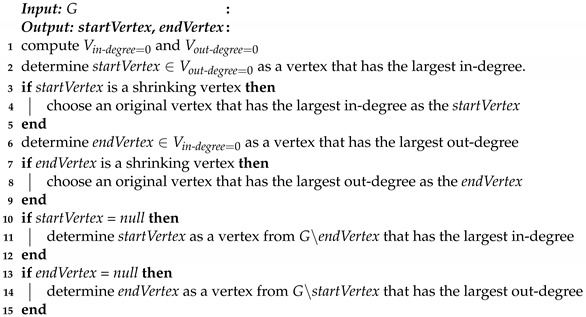


## 6. Evaluation Results

We use the ATC (Asia and Pacific Trade Center) pedestrian group data set [38] to evaluate the effectiveness of the proposed approaches. This data set was collected in a shopping center of Osaka, Japan using 36 Panasonic D-IMager, 11 ASUS Xtion PRO structured light cameras, and 2 Velodyne HDL-32E rotating laser scanners. The data include the IDs of the users in the shopping center and their 3D locations, speeds, directions of movement, and face orientations at different timestamps. We first evaluate the performance of the aligned trajectory interpolation approach and then the time deviation estimation and elimination approach.

### 6.1. Evaluation of Aligned Trajectory Interpolation

We first sample the ATC data within a certain period, and regard them to be the sensing data for human group recognition. To simulate different data collection frequencies, the period of data collection follow a normal distribution N (*F*, 0.2). Aligned trajectory interpolation approach is used to synchronize the data. We mainly check the accuracy of synchronized data when matching the real data, and the accuracy of group recognition.

Fr$\overset{´}{e}$chet distance [39,40] is used to measure how accurate the synchronized data match the real data. This measurement is based on dog-man distance measurement model where a person holds a dog by a rope in an arbitrary speed and the distance between them is measured by the length of the rope. It is commonly used to measure the distance of two curves.

**Figure 6:**
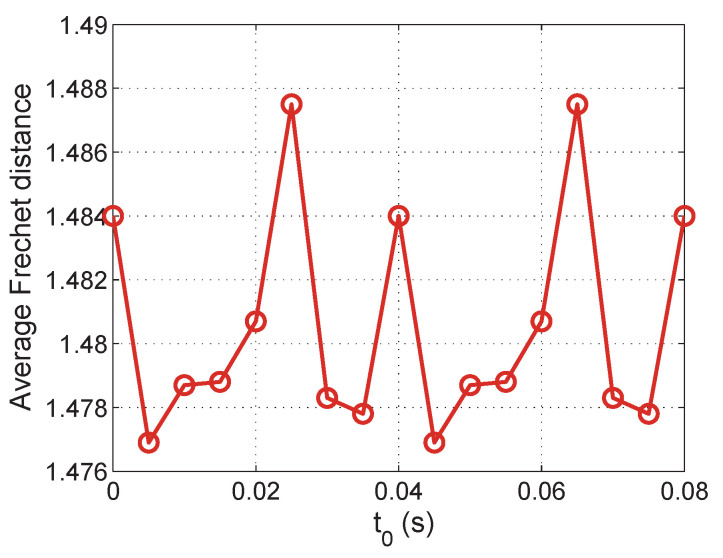
Average Fr$\overset{´}{e}$chet distance with different $t_{0}$.

We first check the Fr$\overset{´}{e}$chet distance between the synchronized data and the real data with different *t${}_{0}$*. The result is shown in Figure 6 where *F* is set to 3s and *f* is set to 0.04s. The average Fr$\overset{´}{e}$chet distance changes periodically with the change of *t${}_{0}$*. Different values of *t${}_{0}$* lead to different Fr$\overset{´}{e}$chet distances. The minimum value is achieved when *t${}_{0}$* equals 0.005, which matches the result of our approach in previous sections.

**Figure 7:**
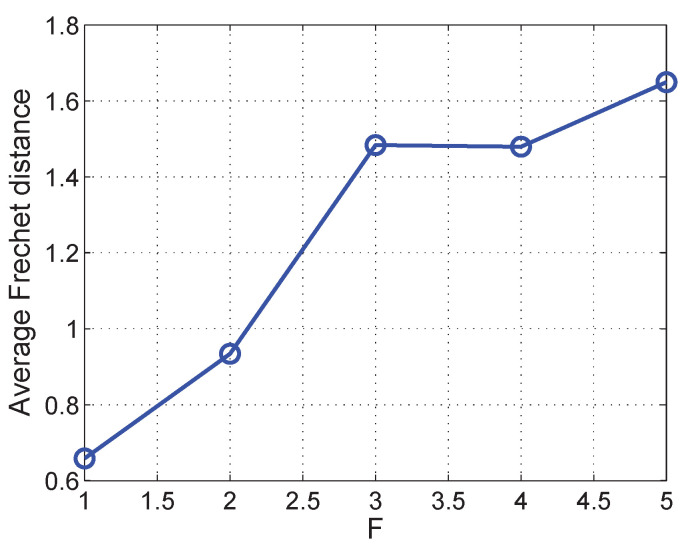
Average Fr$\overset{´}{e}$chet distance with different *F*.

We further check the Fr$\overset{´}{e}$chet distance between the synchronized data and the real data with different values of *F*. *f* is set to 0.04 s. Figure 7 shows the result. The proposed approach is more effective when *F* is small. As *F* increases, the average Fr$\overset{´}{e}$chet distance slightly increases. This is because sparser location data in the trajectory are obtained at a larger *F*, and therefore less real data are collected. However, the aligned trajectory interpolation in our approach alleviate the increase of average Fr$\overset{´}{e}$chet distance.

**Figure 8:**
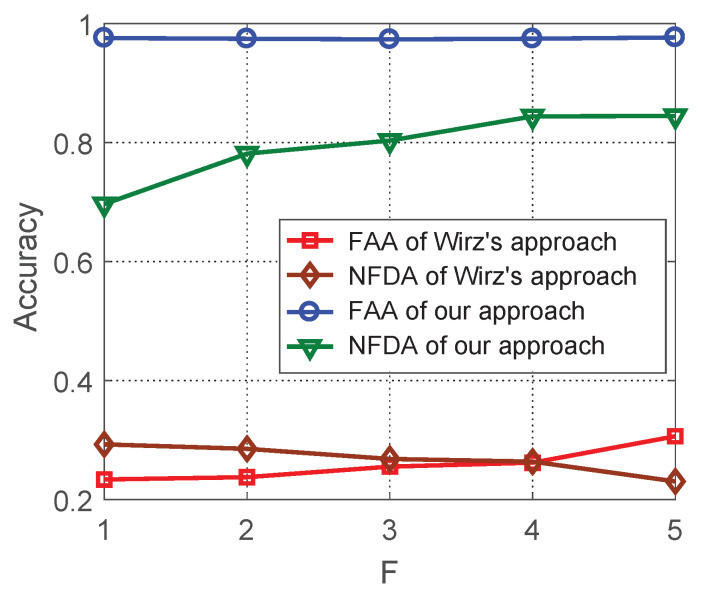
FAA and NFDA of group recognition of different approaches at different *F*.

Finally, we measure the effect of data synchronization on the group recognition. Following the literature [12], FAA(Flock Assignment Accuracy) and NFDA(Number of Flocks Detection Accuracy) are used as the evaluation metrics. The former denotes the normalized average number of objects assigned to correct groups over all timestamps and the latter denotes the normalized number of groups that are correctly identified. When *F* changes from 1 to 5, the result is shown in Figure 8. Firstly, both FAA and NFDA [12] of our approach are higher than that of Wirz et al.’s approach. It validates that our optimized data interpolation helps to improve the accuracy of group recognition. Secondly, FAA of our approach is quite stable and up to 97.7%. This value is even higher than using the original ATC data set, which is 97.4%. This can be explained by the fact that the interpolated trajectory is smoother than the original ATC trajectory by ruling out some location noises. Therefore, our approach applies in a wide range of *F* in different applications.

In the daily life, people’s walking speed is generally at 0.5–2 m/s [10]. If the sampling period is 5 s, the person’s walking distance reaches 2.5–10 m. Even in this difficult situation, the trajectory obtained by using the trajectory interpolation algorithm still performs well in the group recognition. According to the figure, the proposed interpolation approach can achieve up to 97.7% FAA accuracy, and 84.5% NFDA accuracy. When the sampling is more frequent in a normal situation, the performance of our approach will be more desirable.

### 6.2. Evaluation of Time Deviation Estimation

We further validate the effectiveness of the proposed approach to estimate the time deviations among devices. In a system with *n* devices, we assume the time deviation of device *i*, *offset*${}_{i}$ (0<i<n), randomly distributed in (0 s, 15 s). All devices start data collection at the same time and have the same data collection frequency. The transmission time of messages follow a normal distribution *N* (0.1 s, 0.5 s). Initially, we randomly add several message passings among devices, simulating their data communications.

We compare our approach with several existing approaches including random selection, TRandom selection, and hierarchical communication [30]. Random selection randomly requests two devices to perform a message communication. TRandom selection is a random selection based on the shrinking graph generated by Tarjan’s algorithm. The start vertex is selected from $V_{out-degree=0}$ (if it is empty, randomly choose a vertex), and the end vertex is selected from $V_{in-degree=0}$ (if it is empty, randomly choose a vertex). In the hierarchical communication approach, the message exchanges are based a pre-established tree structure. A device is requested to broadcast messages to its children and get responses from them, so that each child device synchronizes to its parent to estimate the time deviation.

**Figure 9:**
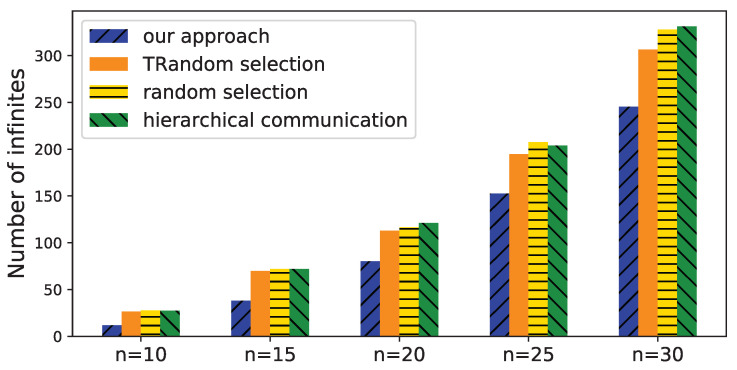
Number of infinites with different number of devices.

We first change the number of devices *n* from 10 to 30 to check the number of *infinites* when estimating their time deviations. The number of initial messages is set to 2n to simulate an unconnected communication structure in the system. The results are shown in Figure 9. When the number of devices increases, the number of *infinites* increases. TRandom selection slightly outperforms random selection because the former combines the strongly connected component of the graph, and hence, avoid invalid messages in it. TRandom selection, random selection, and hierarchical communication show similar performance because they all use a random strategy for adding a new message. Our approach outperforms the other approaches because the messages are optimized to explicitly reduce the number of *infinites*. In a typical scenario where the number of devices is 20, the number of *infinites* of our approach is 71.2% of TRandom selection, 68.9% of random selection, and 66.3% of hierarchical communication.

**Figure 10:**
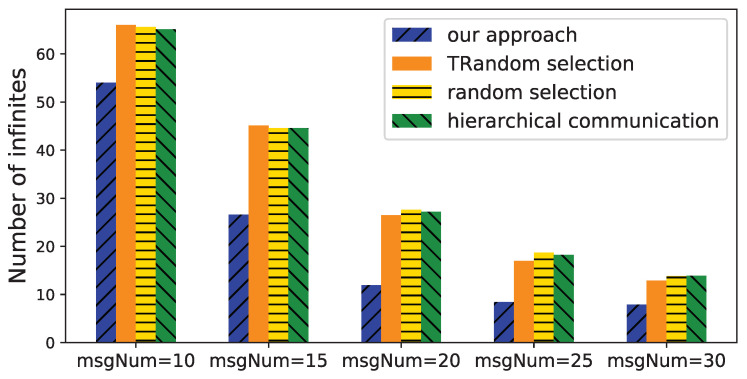
Number of infinites with different number of messages.

We then fix the number of devices to 10 and change the number of messages from 10 to 30 to check the number of *infinites* in the time intervals bounded the time deviations. According to Figure 10, the number of *infinites* decreases with more messages. This is reasonable because more messages likely generate more paths between two devices, and hence, provide a more accurate estimation of the time deviation between them. The reduced number of *infinites* of our approach is much more than that of other approaches. It shows that our approach determines more proper paths to reduce the number of *infinites*, and therefore improves the estimation.

**Figure 11:**
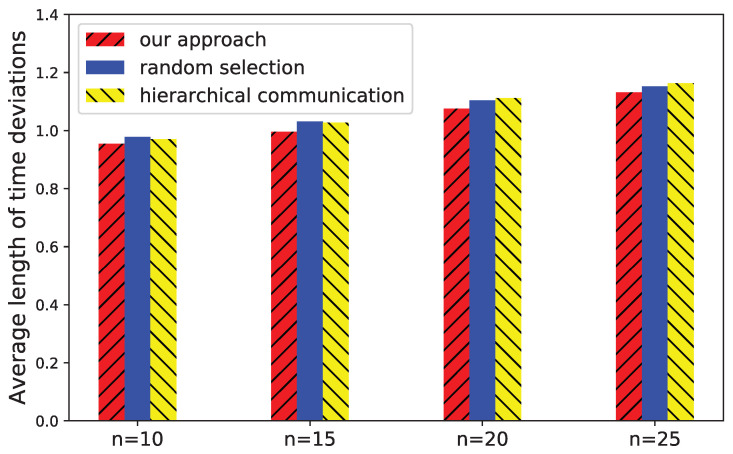
Average length of time deviations with different number of devices.

After that, we compare the approaches when improving the average length of the time intervals bounded time deviations. The result is shown in Figure 11. The number of devices *n* is set to 10 and the initial number of messages is set to 40. In this configuration, the devices are likely to have a strongly connected communication structure. If not, we add a minimum number of extra messages to make the devices strongly connected. In the situation of strongly connected communications, random selection and TRandom selection perform the same, and therefore TRandom selection is not shown in the figure. With the increase of the number of devices, the average length of time intervals of all approaches slightly increases. Our approach outperforms other approaches because it trends to add the messages that probably increase the communication paths between devices. Given same number of messages, our approach leads more accurate estimation of time deviations.

**Figure 12:**
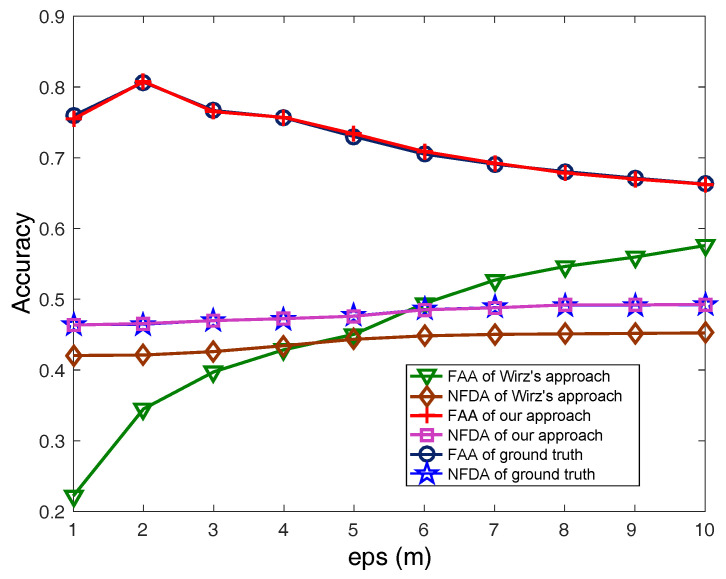
Group recognition accuracy comparison of our approach with other approaches.

Finally, we evaluate the group recognition results of our approach compared with the ground truth and the approach proposed by Wirz et al. [12]. The real data are generated by selecting 91 pedestrians from ATC pedestrian group data set [38] and the collected data are supposed deviated randomly within (−15 s, 15 s) to the real data. The ground truth is based on the real data, and our approach performs time deviation elimination. For the fairness of comparison, we apply the aligned trajectory interpolation algorithm in each time snapshots for all the approaches. In every snapshot, the humans are grouped by their locations. Then, we evaluate the grouping result by the average *FAA* and *NFDA* in the snapshots. In the experiments, we check the average *FAA* and *NFDA* in different values of *eps*, the radius of neighborhood in DBSCAN. The result is shown in Figure 12. Compared with that of the ground truth, the *FAA* and *NFDA* when using Wirz et al.’s approach decrease a lot. It validates that the time deviations affect the group recognition accuracy. The *FAA* and *NFDA* when using our approach are close to the ground truth, which validates the effectiveness of our proposed estimation and elimination approach.

## 7. Conclusions

This study investigated the synchronization of data collection in human group recognition. The challenges for this is that data collection usually has different start time and frequency, and the inherent time deviations of different devices exist. We propose a trajectory interpolation algorithm to synchronize the start time and frequency by minimizing our proposed error function. Moreover, we propose an approach to estimate the time deviations among devices by using message passing. The evaluation results validate the effectiveness of the proposed approach.

In the future, we plan to study how to utilize the messages in the application layer to estimate the time deviations among devices. The time deviation estimation result also can be used to adjust the devices’ data collection frequencies for better synchronization.

## Figures and Tables

**Table 1:** Changes of estimated time deviation with number of messages.

Number of Messages								
(*InfNum*, *AveLength*)	10	15	20	25	30	35	40	45
Exp.1	(8, 1.015)	(9, 1.113)	(8, 1.028)	(0, 1.346)	(0, 0.965)	(0, 0.935)	(0, 0.781)	(0, 0.636)
Exp.2	(12, inf)	(5, 0.607)	(7, 0.616)	(3, 0.965)	(3, 1.314)	(0, 1.036)	(0, 0.941)	(0, 0.705)
Exp.3	(15, inf)	(3, 1.979)	(7, 0.621)	(3, 1.273)	(1, 1.417)	(0, 0.649)	(0, 1.087)	(0, 0.657)
Exp.4	(12, inf)	(10, inf)	(2, 0.944)	(0, 1.531)	(0, 1.414)	(1, 0.925)	(0, 0.93)	(0, 0.489)
Exp.5	(12, 1.342)	(9, 0.795)	(4, 1.317)	(4, 1.231)	(2, 0.594)	(0, 1.281)	(0, 0.74)	(0, 0.495)
Average	(11.8, inf)	(7.2, inf)	(5.6, 0.905)	(2.2, 1.269)	(1.4, 1.141)	(0.2, 0.965)	(0, 0.896)	(0, 0.596)

## Data Availability

Not applicable.
